# DS16570511 is a small-molecule inhibitor of the mitochondrial calcium uniporter

**DOI:** 10.1038/cddiscovery.2017.45

**Published:** 2017-07-17

**Authors:** Naohiro Kon, Michiko Murakoshi, Aya Isobe, Katsuji Kagechika, Naoki Miyoshi, Takahiro Nagayama

**Affiliations:** 1 Medical Science Department, Daiichi Sankyo Co., Ltd., Tokyo, Japan; 2 Biological Research Department, Daiichi Sankyo RD Novare Co., Ltd., Tokyo, Japan; 3 Research Function, Daiichi Sankyo Co., Ltd., Tokyo, Japan; 4 End-Organ Disease Laboratories, Daiichi Sankyo Co., Ltd., Tokyo, Japan; 5 Cell Therapy Laboratories, Daiichi Sankyo Co., Ltd., Tokyo, Japan

## Abstract

In cardiac myocytes, regulation of mitochondrial Ca^2+^ is important for cellular signaling and cardiac contraction. Ca^2+^ entry into the mitochondria is mediated by a highly selective Ca^2+^ channel called the mitochondrial calcium uniporter, which consists of a pore-forming subunit MCU and regulatory subunits such as MICU1. Although pharmacological regulation of the mitochondrial Ca^2+^ influx is a promising approach to controlling the cellular functions, a cell-permeable and specific inhibitor of the mitochondrial calcium uniporter has not yet been developed. Here, we identify a novel cell-permeable inhibitor of the uniporter by a high-throughput screening of 120 000 small-molecule compounds. In our study, DS16570511 dose-dependently inhibited serum-induced mitochondrial Ca^2+^ influx in HEK293A cells with an IC_50_ of 7 *μ*M. DS16570511 inhibited Ca^2+^ uptake of isolated mitochondria from human cells, rat heart and pig heart. Overexpression of hMCU or hMICU1 in HEK293A cells increased mitochondrial Ca^2+^ influx, and the increases were completely suppressed by the pretreatment with DS16570511. DS16570511 also blocks mitochondrial Ca^2+^ overload in a Langendorff perfused beating rat heart. Interestingly, DS16570511 increased cardiac contractility without affecting heart rate in the perfused heart. These results show that DS16570511 is a novel cell-permeable inhibitor of the mitochondrial calcium uniporter and applicable for control of the cardiac functions.

## Introduction

Mitochondria store large amounts of Ca^2+^ for cellular Ca^2+^ homeostasis and regulation of cytosolic Ca^2+^ signaling.^1,2^ Mitochondrial membrane potential generated by the electron transport chain is the driving force of the Ca^2+^ uptake.^3,4,5^ The Ca^2+^ influx into the mitochondria is mediated by an inward-rectifying, highly selective Ca^2+^ channel called the mitochondria calcium uniporter. Although molecular components of the uniporter have been completely unknown for a long time, recent studies have revealed that the *Mcu* gene encodes a channel-forming unit of the uniporter. MCU is a mitochondrial inner membrane protein, and the multimer forms a Ca^2+^ channel in the lipid bilayer.^6^ In addition, the uniporter activity is positively or negatively tuned by some regulatory subunits such as MICU1.^7,8,9,10^ MICU1 is a calcium-binding EF hand-containing protein that regulates the uniporter activity in a Ca^2+^ concentration-dependent manner. MICU1 functions as a gatekeeper of the uniporter by inhibiting MCU activity at resting Ca^2+^ levels, whereas it has a stimulatory role in agonist-challenged cultured cells.^11^ At present, the regulatory mechanism of the uniporter activity is still under discussion.

The identification of the genetic component of the mitochondrial uniporter has provided new opportunities to understanding the biological roles of mitochondrial Ca^2+^ regulation.^1,2,10, 12,13,14,15^ For example, in isolated cardiomyocytes, siRNA-mediated knockdown of *Mcu* enhances peak levels in cytosolic Ca^2+^ oscillation, which results in an increase of muscle contraction.^16^ In pathological aspects such as acute ischemia-reperfusion, cellular injury caused by mitochondrial Ca^2+^ overload is protected by cardiomyocyte-specific knockout of the *Mcu* gene in mice.^14,17^ Importantly, recent human genetic study has shown that mutation of *MICU1* causes brain and muscle disorders.^18^ This study showed that fibroblasts of patients exhibited increased mitochondrial Ca^2+^ influx. Therefore, excess Ca^2+^ influx into mitochondria is toxic for cells and tissues in both acute and chronic pathological situations.

The physiological and pathological evidences suggest that inhibition of the mitochondrial calcium uniporter activity is a novel and unique approach to controlling the cellular functions or to treating mitochondrial diseases. The most well-known inhibitors of the mitochondrial calcium uniporter are the polycationic compounds, Ruthenium Red (RuR) and Ruthenium 360 (Ru360). The Ca^2+^ channel activity mediated by recombinant MCU multimers in the lipid bilayer is inhibited by RuR, showing that RuR directly acts on MCU to inhibit the channel activities.^6^ Although RuR potently inhibits the uniporter activities, its pharmacological use is limited owing to two factors: (1) the impermeability of the plasma membrane and (2) its unspecific inhibitory action against various ion channels.^6^ Therefore, cell-permeability and identifying the specific inhibitor of the uniporter are very important in pharmacology; although, no specific chemical uniporter inhibitor has yet been reported.

In the present study, we performed a high-throughput screening for small-molecule inhibitors of the mitochondrial Ca^2+^ influx in HEK293A cells. We found that DS16570511 blocked the Ca^2+^ influx in the cultured cells and isolated mitochondria. DS16570511 blocked the MCU- or MICU1-dependent increases of Ca^2+^ influx. Isolated perfused heart experiments revealed that the novel inhibitor has an inotropic effect in addition to the protective effect against mitochondrial Ca^2+^ overload.

## Results

### DS16570511 is a novel cell-permeable inhibitor of mitochondrial Ca^2+^ influx

To obtain cell-permeable inhibitors of the mitochondrial calcium uniporter, we generated a human cell-based assay for high-throughput screening (HTS). HEK293A cell lines expressing the mitochondria-targeted Ca^2+^ indicator protein aequorin were established to detect dynamic mitochondrial Ca^2+^ influx. By using the cell-based assay as the first screening system, we screened 120 000 small-molecule compounds. The screening flow of this exploratory project is depicted in Supplementary Figure S1, and five criteria were set to obtain hit compounds: (1) IC_50_ of the hits was to be below 10 *μ*M in the cell-based mitochondrial Ca^2+^ influx assay. (2) IC_50_ in the mitochondrial assay was to be at least sevenfold smaller than the IC_50_ in a counter assay using cytosol-targeted aequorin (this is an indicator of specificity to the mitochondrial calcium uniporter). (3) The inhibitory activity was also to be observed in isolated mitochondria from the human cells. (4) The inhibition was also to be observed in mitochondria prepared from small and large animals (in this case rats and pigs). (5) The inhibitory activity was to be reproducibly observed in the resynthesized compound. We found that DS16570511 was the only one of the hit compounds meeting all of these criteria (Supplementary Figures S2 and S3).

**Figure 1:**
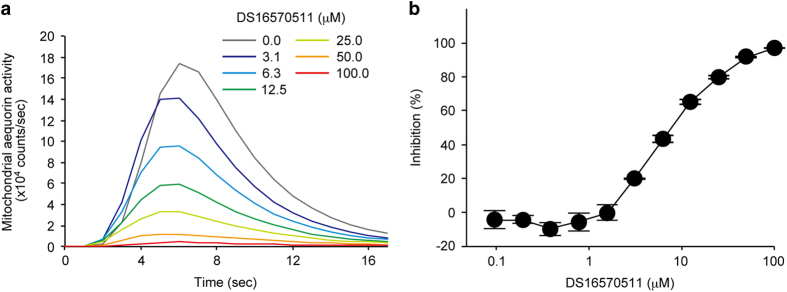
DS16570511 inhibits mitochondrial Ca^2+^ influx in HEK293A cells. (**a**) Representative raw data of serum-induced mitochondrial Ca^2+^ influx in HEK293A cells. (**b**) Effect of DS16570511 on serum-induced mitochondrial Ca^2+^ influx. The area under the curve of temporal aequorin activities is used for calculation of inhibitory activities. Inhibition 0 or 100% is defined as a value of serum-untreated cells or serum-treated cells, respectively. Data are mean with S.E.M. from four independent samples.

In the first screening using the cell-based assay, application of 10% fetal bovine serum to the cells triggered a rapid increase of mitochondrial Ca^2+^ level (Figure 1a). Pretreatment of the cells with DS16570511 inhibited the serum-induced mitochondrial Ca^2+^ influx with an IC_50_ of 7 *μ*M (Figures 1a and b). In order to eliminate broad channel inhibitors or chemicals affecting the aequorin-based detection system, a counter assay was carried out using HEK293A cells expressing aequorin in cytosol. In the assay, the concentration of DS16570511 used to decrease the serum-induced cytosolic Ca^2+^ increase by 50% was ~50 *μ*M. These results indicated that DS16570511 is a cell-permeable and selective inhibitor of mitochondrial Ca^2+^ influx in human cultured cells.

### DS16570511 inhibits Ca^2+^ uptake activity of isolated mitochondria

**Figure 2:**
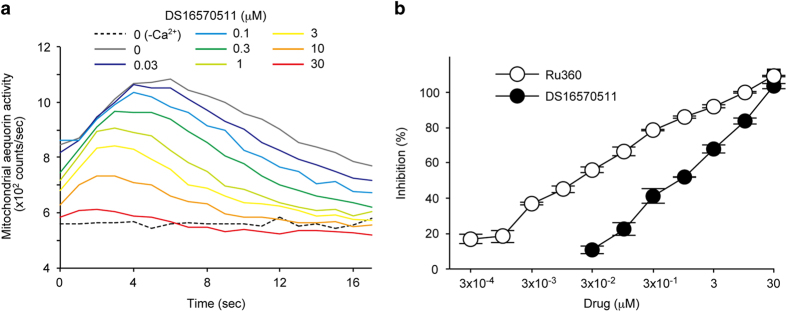
DS16570511 inhibits Ca^2+^ uptake in isolated mitochondria from HEK293A cells. (**a**) Representative raw data of Ca^2+^ uptake in isolated mitochondria. (**b**) Effect of DS16570511 or Ru360 on mitochondrial Ca^2+^ uptake. The area under the curve of temporal aequorin activities is used for calculation of inhibitory activities. Inhibition 0 or 100% is defined as a value of Ca^2+^-untreated cells or Ca^2+^-treated cells, respectively. Data are mean with S.E.M. from three independent samples.

Mitochondrial Ca^2+^ uptake activity is regulated by cytosolic protein kinase signaling,^19^ raising the possibility that HTS hits contained indirect inhibitors of the uniporter, such as inhibitors of cytosolic signaling. To examine the direct inhibitory effect of the compounds on the mitochondrial Ca^2+^ uptake, isolated mitochondria from HEK293A cells were used. Ca^2+^ uptake of the isolated mitochondria was observed as a rapid increase in the luminescence level after application of 100 *μ*M Ca^2+^ (Figure 2a). In the assay, Ru360, a positive control of this assay, inhibited the Ca^2+^ uptake activity with an IC_50_ of 0.02 *μ*M (Figure 2b), and we observed dose-dependent inhibition of the Ca^2+^ uptake by DS16570511 with an IC_50_ of 0.86 *μ*M. These results indicated that DS16570511 directly inhibits the human mitochondrial calcium uniporter.

**Figure 3:**
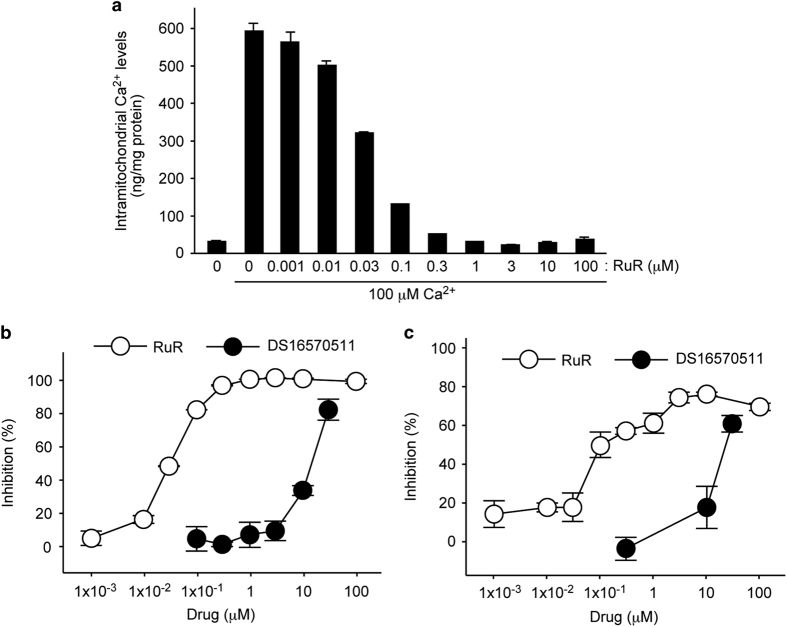
DS16570511 inhibits Ca^2+^ uptake in isolated heart mitochondria. (**a**) Effect of RuR on Ca^2+^ uptake of pig heart mitochondria. *Y* axis shows intramitochondrial Ca^2+^ levels (ng) per 1 mg mitochondrial protein. Data are mean with S.E.M. from three independent samples. (**b**) Inhibition rate of RuR or DS16570511 on Ca^2+^ uptake of pig heart mitochondria. Inhibition 0 or 100% is defined as a value of Ca^2+^-untreated mitochondria or Ca^2+^-treated mitochondria, respectively. Data are mean with S.E.M. from three independent samples. (**c**) Inhibition rate of RuR or DS16570511 on Ca^2+^ uptake of rat heart mitochondria. Data are mean with S.E.M./variation from three or two independent samples for DS16570511 or RuR, respectively.

We then asked whether DS16570511 inhibits Ca^2+^ uptake in isolated mitochondria from animal heart. An atomic absorbance spectrometer-based detection method was employed to analyze intramitochondrial Ca^2+^ levels. We prepared freshly isolated mitochondria from pig heart, and the mitochondria were incubated with 100 *μ*M CaCl_2_. Intramitochondrial Ca^2+^ levels were significantly increased by the Ca^2+^ incubation (Figure 3a). The increase was fully blocked by the pretreatment of positive control RuR with an IC_50_ of 0.03 *μ*M, and DS16570511 blocked the Ca^2+^ uptake activity of the pig heart mitochondria with an IC_50_ of 15 *μ*M (Figure 3b). When mitochondria from rat hearts was used for this assay, the IC_50_s of RuR and DS16570511 were 0.1 and 25 *μ*M, respectively (Figure 3c). These results showed that DS16570511 blocks Ca^2+^ uptake by the isolated mitochondria in both small and large animals. Because the IC_50_ of DS16570511 in the human mitochondria (Figure 2b) was smaller than that of pigs and rats (Figures 3b and c), it is possible that DS16570511 more potently inhibits the uniporter of humans than it inhibits the uniporter of pigs or rats.

### Inhibitory effect of DS16570511 is not dependent on disruption of mitochondrial membrane potential

**Figure 4:**
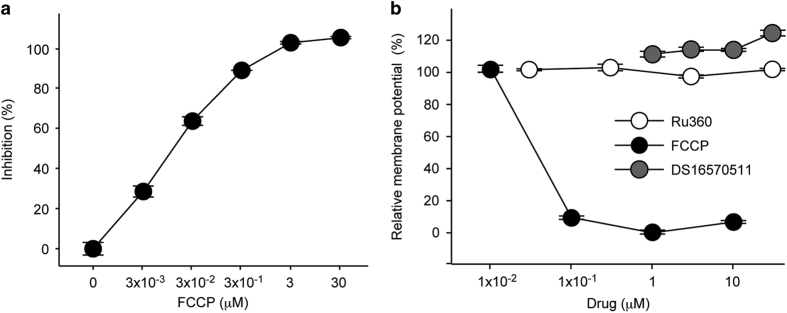
DS16570511 has no effect on mitochondrial membrane potential. (**a**) Effect of FCCP on Ca^2+^ uptake of pig heart mitochondria. (**b**) Effect of FCCP, Ru360 or DS16570511 on membrane potential of pig heart mitochondria. Relative membrane potential 0 or 100% is defined as value of 1 *μ*M FCCP-treated mitochondria or vehicle-treated mitochondria, respectively. Data are mean with S.E.M. from three (**a**) or four (**b**) independent samples.

Next we analyzed the mechanism of action of DS16570511. The previous studies implicated two possible inhibitory mechanisms for the mitochondrial Ca^2+^ uptake: (1) direct blockade of the calcium uniporter and (2) disruption of the membrane potential.^20,21^ Mitochondrial Ca^2+^ uptake is mediated by electrical potential across the mitochondrial inner membrane, which is generated by the oxidative phosphorylation system. Consistent with the previous studies, we observed that carbonyl cyanide-*p*-trifluoromethoxyphenylhydrazone (FCCP), an uncoupler of the mitochondrial membrane potential, significantly inhibited the Ca^2+^ uptake activity of isolated pig heart mitochondria (Figure 4a). To examine effects on the mitochondrial membrane potential, fluorescent indicator JC-10 was used in mitochondria from pig heart. As previously described,^1^ incubation of fresh pig heart mitochondria with FCCP resulted in disruption of the potential in a dose-dependent manner (Figure 4b). On the other hand, Ru360 had no effect on the membrane potential. Therefore, the assay is useful for classifying inhibitors into two types, the uncoupler type compound or the blocker type compound. Importantly, the mitochondrial membrane potential was not reduced by treatment with DS16570511 (Figure 4b), showing that DS16570511 is a blocker type compound like Ru360.

### DS16570511 inhibits MCU- or MICU1-dependent increase of mitochondrial Ca^2+^ influx

**Figure 5:**
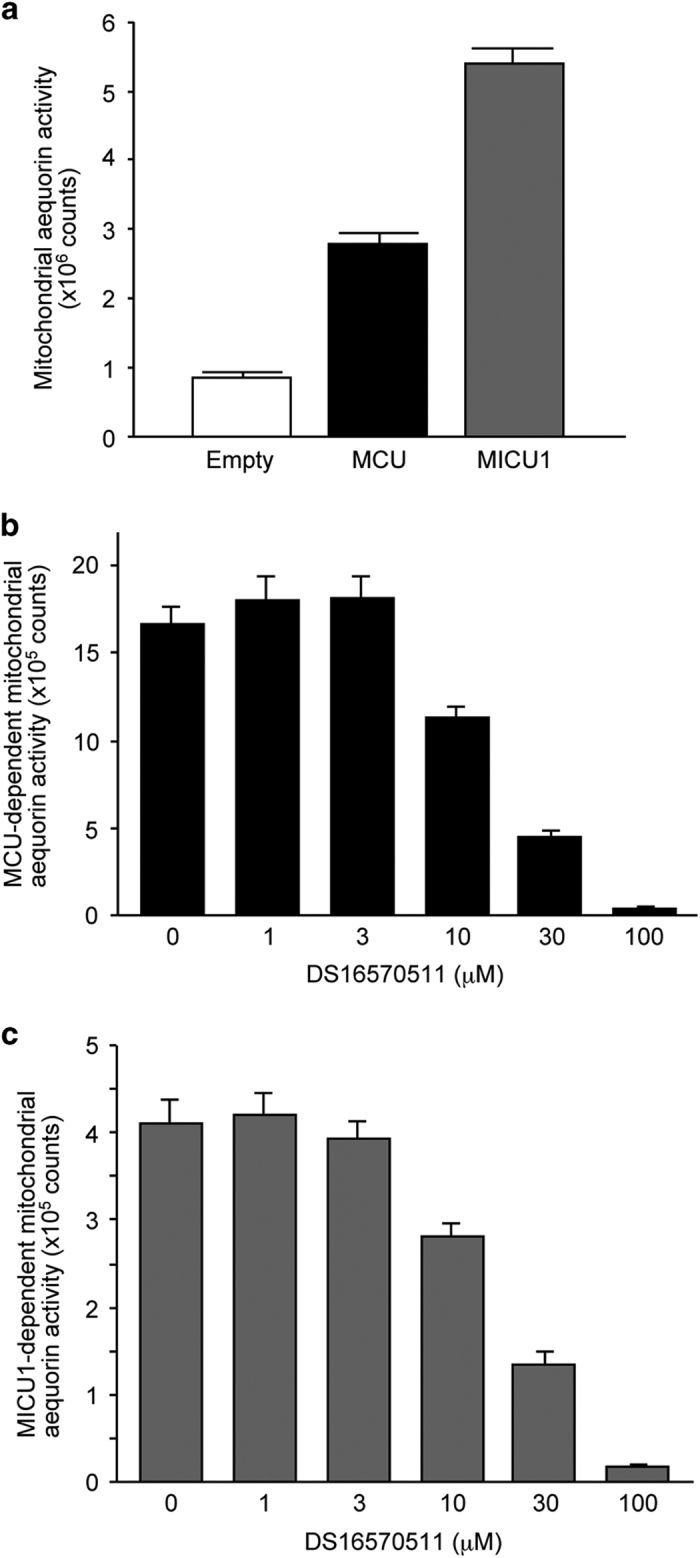
DS16570511 inhibits mitochondrial Ca^2+^ influx by MCU or MICU1. (**a**) Effect of overexpression of MCU or MICU1 on mitochondrial Ca^2+^ influx in HEK293A cells. (**b**) Effect of DS16570511 on MCU-dependent mitochondrial Ca^2+^ uptake in HEK293A cells. (**c**) Effect of DS16570511 on MICU1-dependent mitochondrial Ca^2+^ uptake in HEK293A cells. Data are mean with S.E.M. from eight (**a**) or four (**b** and **c**) independent samples.

Next, to investigate the molecular component of the mitochondrial calcium uniporter, MCU-dependent and MICU-dependent Ca^2+^ influx were examined. Exogenously overexpressed MCU significantly increased mitochondrial Ca^2+^ influx in HEK293A cells (Figure 5a), which was consistent with the previous studies.^8,9^ The MCU-dependent increment of Ca^2+^ influx was blocked by the DS16570511 pretreatment (Figure 5b). We then investigated the effects of the inhibitor on the regulatory subunit MICU1. Previous studies have shown that MICU1 facilitates mitochondrial Ca^2+^ uptake through the presence of a high Ca^2+^ level.^11,22^ Similar to these reports, overexpression of MICU1 significantly increased the serum-induced mitochondrial Ca^2+^ influx (Figure 5a). Interestingly, we found that the MICU1-dependent activation of the uniporter was also blocked by DS16570511 in a dose-dependent manner (Figure 5c). These results showed that DS16570511 blocks both MCU-dependent and MICU1-dependent increases of Ca^2+^ influx.

### DS16570511 blocks mitochondrial Ca^2+^ overload and affects cardiac functions in a rat perfused heart

**Figure 6:**
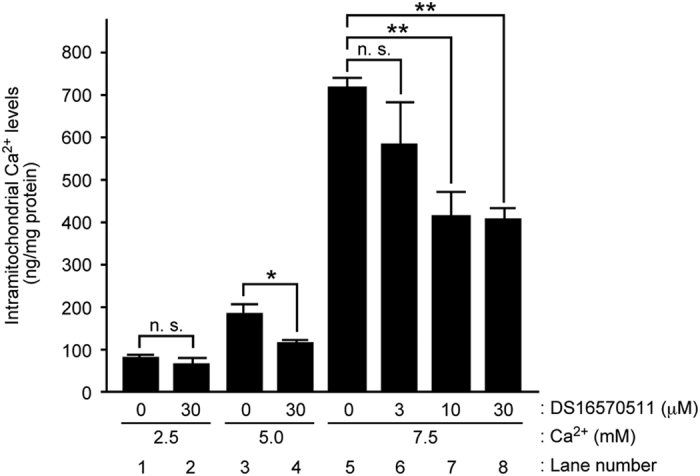
DS16570511 inhibits mitochondrial Ca^2+^ overload in isolated heart. Effect of DS16570511 on mitochondrial Ca^2+^ levels in rat perfused heart. For induction of Ca^2+^ overload in mitochondria *ex vivo*, Ca^2+^ concentration was increased from 2.5 mM to 5 mM or 7.5 mM. The heart was treated with DS16570511 for 10 min before the high Ca^2+^ treatment. Data are mean with S.E.M. from more than three independent samples. Single asterisk shows *P*<0.05, and double asterisks show *P*<0.00005.

We investigated the effect of DS16570511 on mitochondrial Ca^2+^ levels in a rat isolated perfused heart. In the normal condition of the perfusion assay, the Ca^2+^ concentration of the perfusion buffer is 2.5 mM.^23^ To induce mitochondrial Ca^2+^ overload, Ca^2+^ concentration in the perfusion buffer was increased from 2.5 mM to 5 mM or 7.5 mM. The application of a high Ca^2+^ concentration resulted in a dose-dependent increase of mitochondrial Ca^2+^ in the isolated heart (Figure 6, compare lane 1 with lane 3 or 5). In this assay, pretreatment of 30 *μ*M DS16570511 had no significant effect on the Ca^2+^ level in the normal buffer group (Figure 6, compare lane 1 with lane 2). On the other hand, the inhibitor treatment blocked the mitochondrial Ca^2+^ increase induced by 5.0 mM Ca^2+^ buffer (Figure 6, compare lane 3 with lane 4). Furthermore, DS16570511 dose-dependently inhibited the mitochondrial Ca^2+^ overload induced by 7.5 mM Ca^2+^ buffer (Figure 6, compare lane 5 with lanes 6 to 8). These results showed that DS16570511 is applicable to an *ex vivo* working heart for inhibition of mitochondrial Ca^2+^ overload.

**Figure 7:**
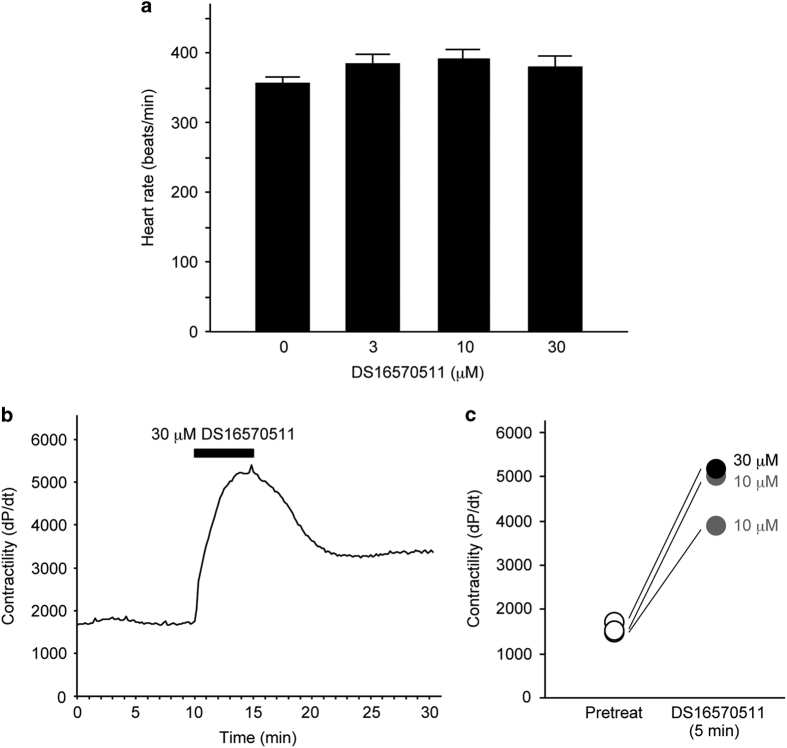
Effects of DS16570511 on cardiac functions. (**a**) Effect of DS16570511 on heart rate in rat perfused heart. Data are mean with S.E.M. from more than four independent samples. (**b**) Representative data showing acute and reversible effect of DS16570511 on contractility. (**c**) Effect of DS16570511 on contractility in rat perfused heart. Plotted are values of pretreatment and 5 min after treatment of 10 or 30 *μ*M DS16570511.

We then investigated the effect of DS16570511 on cardiac functions in the perfused heart. We observed that heart rate was unaffected by DS16570511 in the range of 3–30 *μ*M (Figure 7a). Interestingly, we found that the treatment of DS16570511 increased cardiac contractility in the perfused heart (Figures 7b and c). The increase of contractility was diminished by washout of the drug (Figure 7b), showing that the cardiac effect of DS16570511 is reversible. These results indicated that DS16570511 is a novel inhibitor of the mitochondrial calcium uniporter, which also exerts a positive inotropic action.

## Discussion

Since the discovery of the inhibitory action of RuR against mitochondrial Ca^2+^ uptake, the compound has been used to investigate the relationship between Ca^2+^ entry into the mitochondrial matrix and the function of the organelle.^6^ Recently, several compounds known to exhibit a cellular protective effect have been reported to show inhibitory effects on the uniporter. KB-R7943, originally developed as an inhibitor of plasma membrane Na^+^/Ca^2+^ exchange, was found to inhibit the agonist-induced mitochondrial Ca^2+^ influx in Hela cells.^24^ NecroX-5, one of the derivatives of the reactive oxygen species scavenger NecroX series compounds, was found to attenuate Ca^2+^ accumulation in cultured myocytes.^25^ And it was demonstrated that antibiotic minocycline inhibits Ca^2+^ uptake in isolated rat liver mitochondria.^26^ Original findings of these compounds did not indicate them as being specific inhibitors of the uniporter, and the compounds have additional biochemical actions.^24,27,28^ Therefore, we started exploratory research seeking a small-molecule inhibitor that acts directly against the uniporter by developing novel screening methods. The present study identifies DS16570511 as a cell-permeable and selective inhibitor applicable to a series of experiments using isolated mitochondria, cultured cells and an *ex vivo* perfused heart.

We found that DS16570511 inhibits endogenous activities of mitochondrial Ca^2+^ uptake (Figures 1,Figures 2,Figures 3). In addition, it inhibits Ca^2+^ uptake driven by exogenously expressed MCU or MICU1 (Figure 5). Both components of the uniporter complex have been demonstrated as being essential for mitochondrial Ca^2+^ uptake activity.^2^ Therefore, both MCU and MICU1 are potential binding targets of DS16570511. As the regulatory mechanism of the uniporter activities is still unclear at present, DS16570511 is useful as a novel chemical biological tool for understanding the molecular machinery of the uniporter complex.

In drug development research, it is important to confirm whether biological activities shown by a molecular biological study are also observed in the experiments using a molecularly targeted drug.^29^ Knockdown of *Mcu* by siRNA has been shown to increase contractility in isolated cardiac cells.^16^ Consistent with this study, DS16570511 increased cardiac contractility in the isolated perfused heart (Figure 7). On the other hand, several studies have reported that knockout mice of *Mcu* show no overt baseline phenotype in cardiac functions.^10,13,14,17^ It is possible that acute inhibition such as siRNA-mediated knockdown ^16^ or pharmacological inhibition (Figure 7) is important to the exerting of the inotropic action on the heart.

Mitochondrial Ca^2+^ influx is important to the controlling of cell death events. Inhibition of MCU protects mouse neurons from NMDA receptor-dependent excitotoxicity.^30^ Recent studies have demonstrated that cardiac specific knockout of MCU shows protection against the injury *in vivo.*^14,17^ This is consistent with a number of pharmacological and genetic studies showing potent protective effects of blocking mitochondrial permeability transition in the injury.^31^ Importantly, human genetic study has revealed that mutation of *MICU1* causes mitochondrial Ca^2+^ overload, which promotes the development of brain and muscle disorders.^18^ Because DS16570511 potently blocks both MCU-dependent and MICU1-dependent Ca^2+^ overload (Figure 5), the compound may be an effective treatment for such genetic disorders, as well as for cardiac ischemia-reperfusion injury.

The present study identified a cell-permeable chemical inhibitor of the mitochondrial calcium uniporter and demonstrated that the uniporter activity is adjustable by the compound in the intact tissue. DS16570511 is a potential lead compound and its further study should serve to open up new avenues to satisfying unmet medical needs in mitochondrial diseases.

## Materials and methods

### Animals and reagents

Nine-week-old male Wistar rats were purchased from Japan SLC (Hamamatsu, Japan). All experimental procedures were performed in accordance with the in-house guidelines of the Institutional Animal Care and Use Committee of Daiichi Sankyo. The animals received a standard laboratory diet and filtered water *ad libitum* under specific pathogen-free conditions. Every effort was made to minimize animal suffering and to reduce the number of animals employed. All animal studies were also conducted in accordance with the ARRIVE guidelines.^32,33^ The following were also purchased: fresh pig hearts from Tokyo Shibaura Zouki Co., Ltd. (Tokyo, Japan); pIRES-puro vector from Clontech Laboratories, Inc. (Mountain View, CA, USA); HEK293A cells, pcDNA3.1, Hanks Balanced Salt Solution, coelenterazine h and a mitochondria isolation kit from Thermo Fisher Scientific Inc. (Waltham, MA, USA); fetal bovine serum and RuR from Sigma-Aldrich, Inc. (St Louis, MO, USA); JC-10 from Enzo Life Sciences, Inc. (Farmingdale, NY, USA).

### Aequorin assay

For dynamic measurements of mitochondrial Ca^2+^ levels in intact cells, HEK293A cells were stably transfected with pIRES-puro vector expressing mitochondria-targeted aequorin.^20^ One day after plating on a 15-cm dish (8×10^6^ cells/dish), the cells were harvested and incubated with 2.5 *μ*M coelenterazine h in aequorin assay buffer (200 mM Hanks Balanced Salt Solution, 25 mM HEPES (pH 7.0) and 0.1% bovine serum albumin) for 2 h at room temperature. The cells (8.1×10^4^ cells/well) were then treated with DS16570511 for 20 min in a 96-well plate at room temperature. For induction of intracellular Ca^2+^, the cells were treated with 10% fetal bovine serum, and luminescence levels were measured by using a Centro LB960 luminometer (Berthold Technologies, Oak Ridge, TN, USA).

In order to evaluate mitochondrial Ca^2+^ uptake activity by MCU or MICU1, HEK293A cells were transiently transfected with the mitochondria-targeted aequorin vector and pcDNA3.1 vector containing human *Mcu* (NCBI Reference Sequence: NM_138357.2) or *Micu1* (NM_001195518.1). One day after the transfection, the luminescence levels were measured as described above. MCU- or MICU1-dependent activity was calculated by subtracting the luminescence level of cells transfected with empty vector from that of cells expressing MCU or MICU1, respectively.

To monitor Ca^2+^ uptake activity of isolated mitochondria, mitochondria were prepared from the cells (1×10^8^ cells) stably expressing mitochondria-targeted aequorin by using a mitochondria isolation kit. The mitochondrial pellet was suspended in 12 ml of swelling buffer (150 mM sucrose, 50 mM KCl, 2 mM KH_2_PO_4_, 5 mM succinic acid and 20 mM Tris (pH 7.4)) containing 2.5 *μ*M coelenterazine h. Five minutes after incubation of the mitochondria solution with DS16570511 in a 96-well plate at room temperature, CaCl_2_ (final concentration of 100 *μ*M) was applied to the solution. The luminescence levels were detected as described above.

### Ca^2+^ uptake assay using isolated heart mitochondria

Mitochondria were isolated by using the mitochondria isolation kit and then dissolved in the swelling buffer. The protein concentration of the mitochondrial solution was 5 mg/ml. Thirty minutes after application of CaCl_2_ (final concentration of 100 *μ*M) to the solution, the mitochondria were collected by centrifugation (3000 *g*) at 4 °C. The pellets were resuspended in the swelling buffer containing 1 *μ*M RuR. After collection of the mitochondria by centrifugation (3000 *g*), the pellets were dried and dissolved by 40 *μ*l of sulfuric acid at 95 °C. The solution was then diluted by water, and Ca^2+^ concentration of the solution was measured by atomic absorbance spectrometer (Hitachi High-Technologies Corporation, Z-2710, Tokyo, Japan).

### Mitochondrial membrane potential assay

Pig heart mitochondria were prepared as described above and suspended in the swelling buffer containing 1 *μ*M JC-10. The protein concentration of the solution was 5 mg/ml. Five minutes after incubation of the mitochondria with DS16570511 at room temperature, fluorescence intensities were measured by a FlexStation 3 (Molecular Devices, LLC, Sunnyvale, CA, USA) using the green channel (excitation/emission wavelength: 485/538 nm) or the red channel (excitation/emission wavelength: 485/612 nm).

### Rat isolated perfused heart assay

The hearts were rapidly excised from 9-week-old male Wistar rats and perfused at 37 °C with Krebs-Ringer perfusion buffer (127.2 mM NaCl, 4.7 mM KCl, 2.5 mM CaCl_2_, 1.2 mM KH_2_PO_4_, 1.2 mM MgSO_4_, 25 mM NaHCO_3_ and 5.5 mM glucose (pH 7.4)) equilibrated with 95% O_2_/5% CO_2_. A balloon was inserted through the left atrium into the left ventricle. Heart rate and contractility were continuously recorded with a data acquisition system (PowerLab, ADInstruments, Bella Vista, NSW, Australia).
