# Supplementary figures and images for: DS16570511 is a small-molecule inhibitor of the mitochondrial calcium uniporter
Source: Cell Death Discov. 2017 Jul 17;3:17045–. doi: 10.1038/cddiscovery.2017.45 (PMC5511861; doi:10.1038/cddiscovery.2017.45)

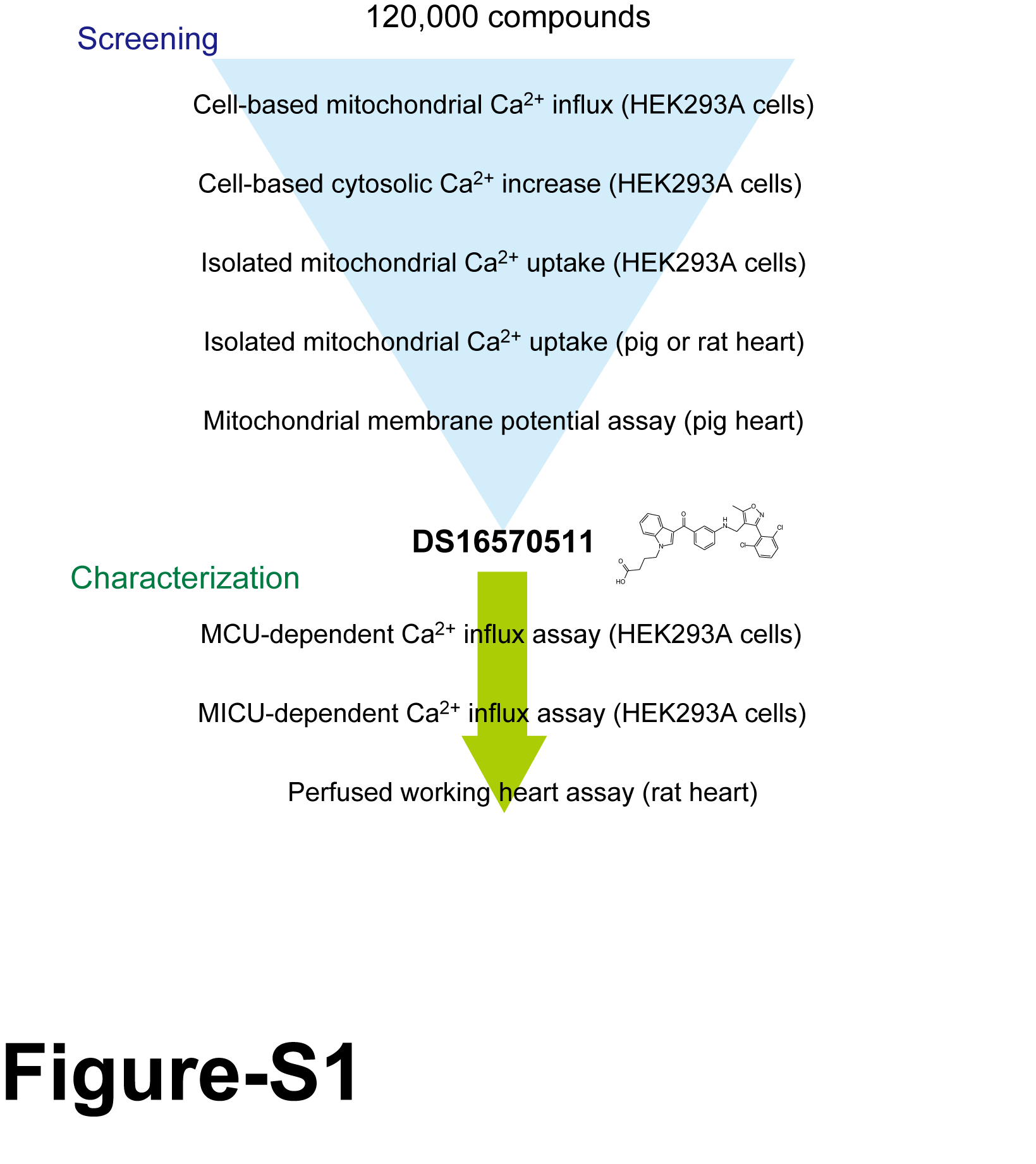

Supplement: Supplementary Figure S1 [file cddiscovery201745-s1.tiff]

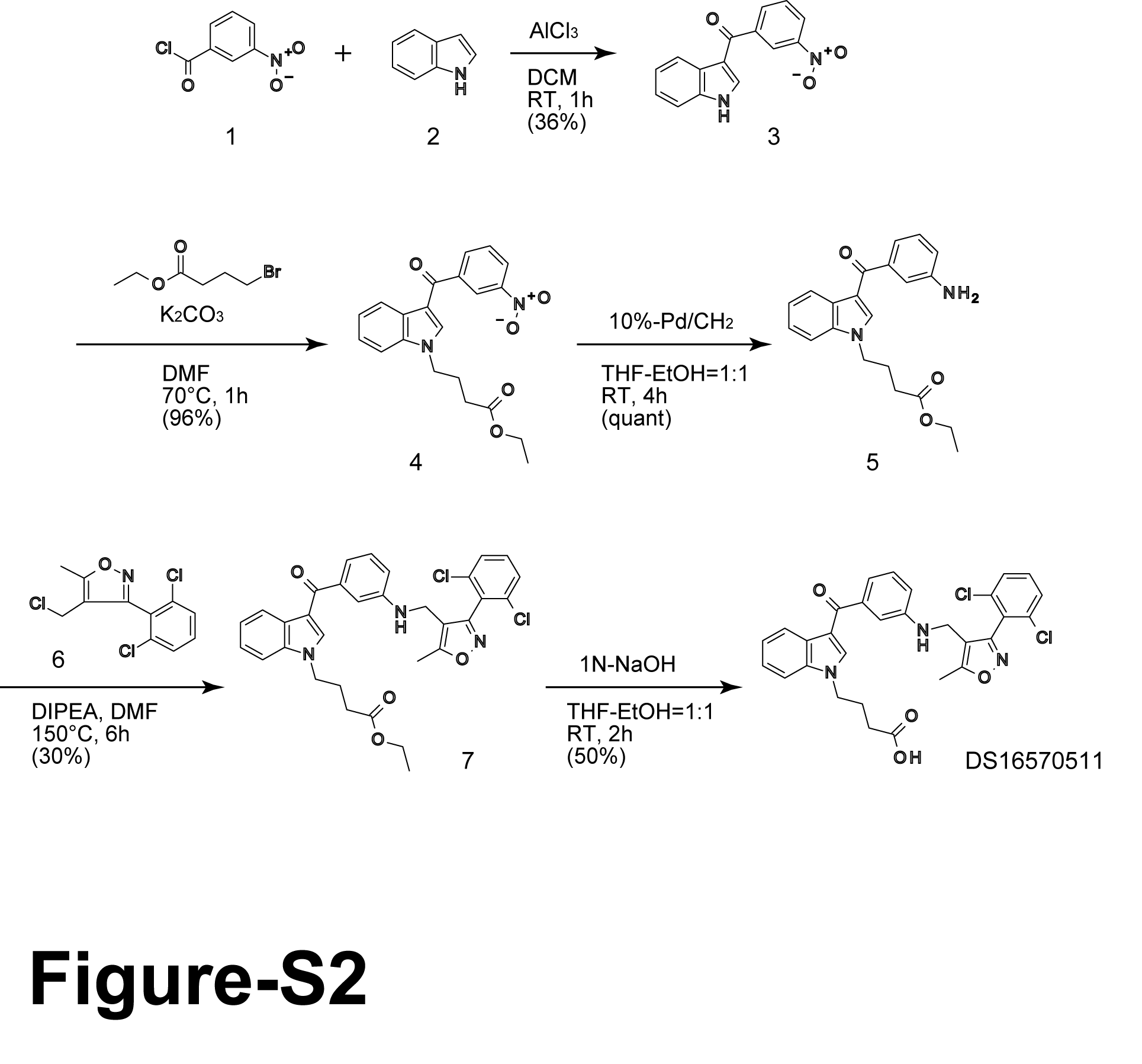

Supplement: Supplementary Figure S2 [file cddiscovery201745-s2.tiff]

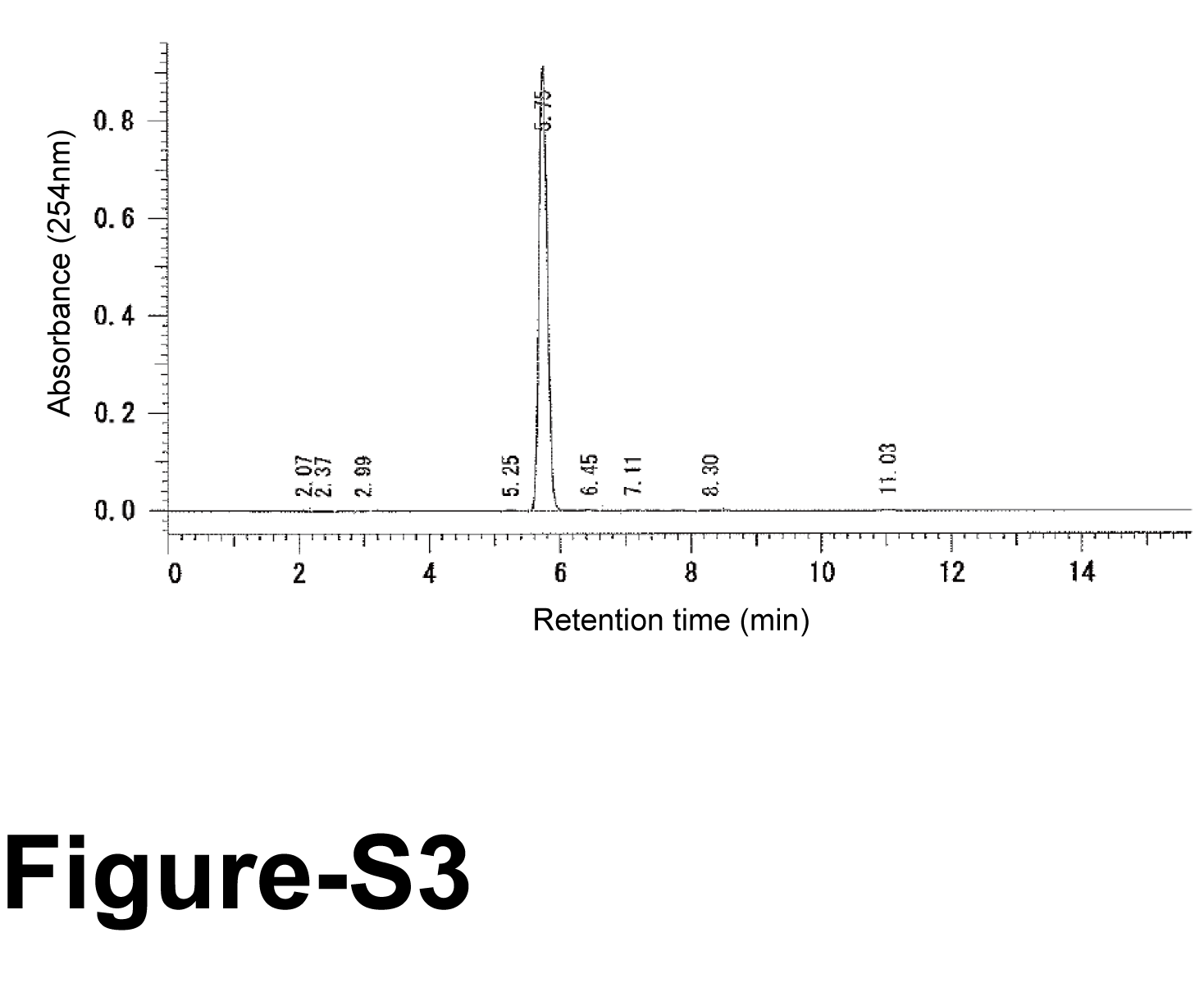

Supplement: Supplementary Figure S3 [file cddiscovery201745-s3.tiff]
