# Supplementary material for: DS16570511 is a small-molecule inhibitor of the mitochondrial calcium uniporter
Source: Cell Death Discov. 2017 Jul 17;3:17045–. doi: 10.1038/cddiscovery.2017.45 (PMC5511861; doi:10.1038/cddiscovery.2017.45)
Supplement: Supplementary Figure Legends [file cddiscovery201745-s4.docx]

**Supplementary figure legends**

**Figure S1 Screening flow for inhibitors of the mitochondrial calcium uniporter.**

For the screening of inhibitors of the mitochondrial calcium uniporter from 120,000 small-molecule compounds, five assay systems were developed. As a result, DS16570511 was identified as a hit compound. Subsequently, DS16570511 was analyzed by three assays for characterization of the properties, MCU-dependency, MICU1-dependency and applicability for *ex vivo* studies.

**Figure S2 Synthetic routes to DS16570511.**

Synthetic method for compound 5 was previously described in below paper.

Sawada K, Okada S, Kuroda A, Watanabe S, Sawada Y, Tanaka H. 4-(Benzoylindolizinyl)butyric Acids; Novel Nonsteroidal Inhibitors of Steroid 5α-Reductase. III. *Chem Pharm Bull* 2001; **49**: 799-813.

**Figure S3 HPLC data of synthesized DS16570511.**

Y-axis shows absorbance at 254 nm. Area of the main peak is > 98% of total area of peaks.
